# Supplementary material for: Evaluation of the QoS policy model of an ordinary 5G smart city cluster with predominant URLLC and eMBB traffic
Source: PLoS One. 2023 Dec 8;18(12):e0295252. doi: 10.1371/journal.pone.0295252 (PMC10707587; doi:10.1371/journal.pone.0295252)
Supplement: Attachment — Submitted filename: Responses.doc [file pone.0295252.s002.doc]

**Response to the Academic Editor comments**

Dear Academic Editor,

on behalf of the team of authors, I thank you for the time devoted to the article and the rational comments made. All your comments, as well as the comments of respected Reviewers, were taken into account in the revised version of the article.

I sincerely hope that you will consider what has been done sufficient to support the publication of the article.

Sincerely, Viacheslav Kovtun.

1) *All acronyms must be written in full the first time they are used within text*

I took this remark into account everywhere except the title. If I expand the abbreviations into the title, it will turn out to be too cumbersome: “Evaluation of the Quality of Service Policy Model of an Ordinary 5G Smart City Cluster with Predominant Ultra-Reliable Low-Latency Communications and enhanced Mobile BroadBand Types of Traffic”. I hope you agree with this sentiment.

2)"...*The results of the comparison testify in favour of the superiority of the author's approach..." Give a summary of the obtained results to support the above claim*.

I have modified the ending of the Summary following this remark, dear Colleague. However, I will note that in Section 3, the comparison of QoS model instances is made using a complex, multi-valued metric. Mention of any one indicator will be incomplete from the point of view of the result obtained. Mentioning all indicators means making the Summary too massive. Please take this fact into account. A detailed and detailed analysis is carried out in the Discussion section because that is what it is intended for.

3) *Just before the "2 Materials and Methods" section, give an outline on how this paper is organized*.

Done, dear Colegue.

4) *"...The focus of our study will be the process..." Change the above to "..The focus of our study was the process..."*

Done, dear Colleague. Thank you.

5) *Ensure that all the presented figures are appropriately interpreted and discussed*.

Checked, dear Colleague.

**Response to the Reviewer #1 comments**

Dear Colleague,

I am sincerely grateful to you for the time you devoted to the article and the positive reception of this research. I carefully revised the text of the article following your recommendations. I hope you will support the current version of the article.

With respect,

on behalf of the team of authors,

Viacheslav Kovtun.

1- *The abstract did not mention the adopted standards or their numerical values that confirm the superiority of the proposed approach over other methods*.

Thank you, dear Colleague. I changed the ending of the Abstract to look like this:

Also, in the proposed qualitative metric, a comparison of the author's concept with a parametrically close analogue (the well-known QoS policy scheme, which takes into account the phenomenon of reservation of communication resources), determined taking into account the reservation of communication resources, was made. The results of the comparison testify in favour of the superiority of the author's approach in the proposed metrics.

Please note that the Discussion section is devoted to a detailed analysis of the superiority of the proposed scheme. I don't want to overload the Abstract with too much detailed information.

2- *Unify the format of keywords by writing them all with the first capital letter*

Done, dear Colleague.

3- *In the introduction, the researcher does not explain what failures previous research has suffered from, or how the research presented here excelled in relation to them. In addition, the structure of the research was not explained in a paragraph at the end of the introduction*

Thank you for this remark, dear Colleague. I have added a critical generalization to the mentioned analogues:

In all the works mentioned above, the authors either view the 5G cluster as a closed system or do not take into account the features of the simultaneous operation of URLLC and eMBB technologies on the 5G platform. At the same time, the closest analogues to this research are works [28, 29]. A feature of the works [28, 29] is the use of queuing systems with random requirements for modelling URLLC-eMBB interactions. At the same time, we note that the authors of the mentioned studies try to abstract from the conditions in which the 5G cluster will operate. Thus, while increasing the universality of the results obtained, the proposed solutions lose adequacy for specific objects. We should also pay attention to the fact that the authors formulate their models [26-28] in a well-looking, but cumbersome mathematical basis, which does not allow the methods obtained to be used to analyze real 5G clusters with a large amount of communication resources. In this article, we focus on addressing these limitations.

At the end of Section 1, I provided information about the structure of the article:

The article is organized as follows. Section 2 presents the main scientific result of the study. In particular, the research was set up, including the formation of the main parametric space of the model of the studied process. Next, a qualitative metric is formulated for evaluating an arbitrary instance of the studied process. Exact and approximate methods are formulated for the evaluation of the QoS policy model of an ordinary 5G smart city cluster (including some typical cases). In Section 3, from the standpoint of mathematical statistics and the experiment planning theory, the results of evaluating an instance of a QoS policy model of an ordinary 5G smart city cluster are presented, both using the author’s approach and in comparison with an analogue. Section 4 analyzes the obtained empirical results. Section 5 draws general conclusions on the work and formulates directions for promising further research.

4- *Standardize the format of references*

Done, dear Colleague.

**Response to the Reviewer #2 comments**

Dear Colleague,

I am sincerely grateful to you for the time you devoted to the article and the positive reception of this research. I carefully revised the text of the article following your recommendations. I hope you will support the current version of the article.

With respect,

on behalf of the team of authors,

Viacheslav Kovtun.

1- *What is the problem to be solved and the existing challenges should be explained in the abstract*.

Thank you for the advice, dear Colleague. I took it into account by adding the following phrase to the Abstract:

A typical element of the smart city’s information and communication space is a 5G cluster, which is focused on serving both new and handover requests because it is an open system. In an ordinary 5G smart city cluster, Ultra-Reliable Low-Latency Communications (URLLC) and enhanced Mobile BroadBand (eMBB) traffic types prevail. The formation of an effective QoS policy for such an object (taking into account the potentially active slicing technology) is an urgent problem.

2- *The key comparison values should be mentioned in the abstract. These values show the efficiency of the proposed method*.

Thank you, dear Colleague. I changed the ending of the Abstract to look like this:

Also, in the proposed qualitative metric, a comparison of the author's concept with a parametrically close analogue (the well-known QoS policy scheme, which takes into account the phenomenon of reservation of communication resources), determined taking into account the reservation of communication resources, was made. The results of the comparison testify in favour of the superiority of the author's approach in the proposed metrics.

Please note that the Discussion section is devoted to a detailed analysis of the superiority of the proposed scheme. I don't want to overload the Abstract with too much detailed information.

3- *In introduction, “URLLC technology is designed to support mission-critical communications with low latency, high security and reliability. Finally, eMBB technology aims to provide high data transfer rates.”, please add proof to this claim*.

Dear Colleague, you rightly drew attention to this interesting point. I cannot answer this question in a few words. A fairly detailed answer is in my article <https://doi.org/10.1038/s41598-022-20135-5> (Reference no. 2) With that said, I've changed the piece of text you mentioned:

The URLLC technology is designed to support mission-critical communications with low latency, high security and reliability. Finally, eMBB technology aims to provide high data transfer rates. This issue is discussed in more detail in [2].

4- *In introduction, “To a first approximation, this space can be segmented into industrial, specialized and civil clusters that require wireless communication coverage.”, What was this segmentation based on*?

Dear Colleague, the criterion for the mentioned segmentation is the destination of the majority of the traffic, which is controlled by the base station, which is a formative element of the corresponding geographical location in information and communication space. For example, if there is a big supermarket in the base station coverage sector, then this cluster can be classified as “civil”.

5- *In introduction, “At the same time, typical information exchange in such a 5G cluster requires the implementation of URLLC (ensuring the information needs of critical infrastructure and institutions) and eMBB (ensuring the information needs of citizens and the service sector) technologies.”, Please add a reference to support this statement*.

Done, dear Collegue.

At the same time, typical information exchange in such a 5G cluster requires the implementation of URLLC (ensuring the information needs of critical infrastructure and institutions) and eMBB (ensuring the information needs of citizens and the service sector) technologies [6].

6- *Adding a recent reference that addresses the challenges of QoS in the introduction and related work. The “Related Work” section lacks of enough references. I strongly recommend that the author improve this section by adding references that support all the claims, the challenges of QoS, and motivation of the problem. The author may precisely and comprehensively point out the current issues and existing solutions. I suggest adding more related reference such as: …*

Thank you for the excellent advice, dear Colleague. It pushed me into a whole direction of further research. Based on this, I added the following paragraph at the end of the Conclusions section:

Also note that we presented the main scientific result of the study in the basis of mathematical statistics, and recovery theory. This approach is characterized by sufficient universality (subject to adequacy control) and low computational complexity. The shortcoming of the proposed approach is that the procedure for adapting the proposed mathematical apparatus to the specific conditions of 5G network deployment cannot be automated. In this context, a promising direction for further research is also the synthesis of models based on artificial intelligence, which can complement the result presented in the article. The authors see works such as [33-37] as analogues for this area of research.

I have added the appropriate links to the References section.

7- *In introduction, “We should also pay attention to the fact that the authors formulate their models in a well-looking, but cumbersome mathematical basis “, the mathematical foundations must be mentioned*.

Dear Colleague, thank you for drawing attention to this phrase. It relates to the analysis of works [26-28], so I mentioned them in the edited version of the sentence, and also attached it in the paragraph above:

A feature of the works [28, 29] is the use of queuing systems with random requirements for modelling URLLC-eMBB interactions. At the same time, we note that the authors of the mentioned studies try to abstract from the conditions in which the 5G cluster will operate. Thus, while increasing the universality of the results obtained, the proposed solutions lose adequacy for specific objects. We should also pay attention to the fact that the authors formulate their models [26-28] in a well-looking, but cumbersome mathematical basis, which does not allow the methods obtained to be used to analyze real 5G clusters with a large amount of communication resources. In this article, we focus on addressing these limitations.

8- *The article did not address the research gap*.

Dear Colleague, in response to this remark, I changed the aim of the research as follows:

The aim of the research is formulated as follows: to create a computationally efficient, holistic concept for evaluating an arbitrary instance of the QoS policy of an ordinary 5G smart city cluster with predominant URLLC and eMBB traffic, which will make it possible to close the research gap identified as a result of the analysis of analogues.

9- *It also did not explain sufficiently how to achieve the objectives*.

Thank you, dear Collegue. I added Research objectives this way:

Research ***objectives*** are:

- to determine the parametric space of stable and controlled characteristics that characterize the research object (presented in Section 2.1),

- to formulate a comprehensive qualitative metric for evaluating an arbitrary instance of the implementation of the research object (presented in Section 2.2),

- to formulate an accurate parameterized concept for evaluating an arbitrary instance of the QoS policy of an ordinary 5G smart city cluster (presented in Section 2.3),

- to formulate a computationally efficient approximate parameterized concept for evaluating an arbitrary instance of the QoS policy of an ordinary 5G smart city cluster (presented in Section 2.3),

- to analytically investigate partial cases of QoS policy implementation of an ordinary 5G smart city cluster in terms of an approximate evaluation concept (presented in Section 2.4),

- justify the adequacy of a mathematical apparatus proposed and demonstrate its functionality in the context of a research aim (presented in Section 3 and discussed in Section 4).

10- *The symbols mentioned in the statement of research are not defined*.

Dear Colleague, I have carefully re-read and supplemented the text of Section 2.1. All entered variables and abbreviations are described and deciphered in the current version of the article.

11- *In statement of research, “We will ensure the balance of the QoS policy under the following conditions:”, What was relied upon in presenting these conditions*?

Dear Colleague, these conditions are the minimum sufficient to describe the proposed concept of interaction between URLLC and eMBB requests in a 5G smart city cluster, which we consider an open system (this is taken into account by introducing the handover class of requests).

12- *In 2.2, “The proposed approach to evaluating the QoS policy of an ordinary 5G smart city cluster is accurate.”, What is the proof*?

Dear Colleague, all the correctness of the mathematical apparatus provided in Section 2 is confirmed by its subsequent formation with appropriate comments, as well as the correctness of the application of mathematical operations that are described in such areas of knowledge as recovery theory, and Markov process theory. Ultimately, the adequacy and functionality of the proposed mathematical apparatus are proven empirically in Section 3, taking into account the provisions of mathematical statistics, which is provided for by the experiment planning theory.

13- In 2.3, “At the same time, we take into account that URLLC connections are short-lived compared to eMBB connections”, please add a reference as proof.

Done, dear Collegue:

At the same time, we take into account that URLLC connections are short-lived compared to eMBB connections [2], however, the share of active URLLC connections in the traffic of the studied 5G cluster is dominant.

14- *In 2.3, “After analyzing expression (12), we conclude that a stationary probability distribution of a model F states with a state space i F is identical”, the explanation must be detailed regarding identical*.

Thank you. The corresponding link is added:

After analyzing expression (12), we conclude that a stationary probability distribution of a model $\tilde{F}$ states with a state space $F_{i}$ is identical to the stationary probability distribution of an Erlang model of type $M/M/C-i/0$ [32] states with state-dependent intensities of an arrival of new requests and an intensity $\mu_{\Sigma u}$ of serving received requests by a single device.

15- *In 2.4, What is the effect of C on QoS*?

Thank you for your question, dear Colleague. Please note the second paragraph of Section 2.1:

Suppose that the channel resource of an ordinary 5G cluster includes  units, …

16- *In 2.4, “If the inequality he u T +T  C is not fulfilled, then we proceed to Stage 3, otherwise, we proceed to Stage 4.”, why*?

Dear Colleague, in the information technology you mentioned, Stage 2 characterizes the situation when the studied 5G cluster has enough resources to service the incoming request, and Stage 3 – when there are not enough resources. An indicator of the transition to the optional Stage 3 is the inequality $T_{he}+T_{u}\leq C$, formulated in relation to the parameter $C$ mentioned above.

17- *In result section, “QoS1 : C =160 , 140 u T = , 120 he T = , 10 u  = , 6 hu  = , 4 e  = , 3 he  = , 10 u   = , 2 e   = .”, How were these values established*?

Dear Colleague, these parameters were obtained as a result of analyzing long-term statistics from the Information Technology Department of the City Council of my hometown Vinnytsia (Ukraine). As you understand, in wartime there is no point in focusing too much attention on the source of data in this case. Unfortunately, war takes both lives and freedom.
